# Rab11a promotes proliferation and invasion through regulation of YAP in non-small cell lung cancer

**DOI:** 10.18632/oncotarget.15359

**Published:** 2017-02-15

**Authors:** Qianze Dong, Lin Fu, Yue Zhao, Yaming Du, Qingchang Li, Xueshan Qiu, Enhua Wang

**Affiliations:** ^1^ Department of Pathology, First Affiliated Hospital and College of Basic Medical Science, China Medical University, Shenyang, China; ^2^ Department of Cardiovascular Thoracic Surgery, The First Affiliated Hospital of Jinzhou Medical University, Jinzhou, China

**Keywords:** Rab11a, Hippo, YAP, non-small cell lung cancer, proliferation

## Abstract

Rab11a, an evolutionarily conserved Rab GTPases, plays important roles in intracellular transport and has been implicated in cancer progression. However, its role in human non-small cell lung cancer (NSCLC) has not been explored yet. In this study, we discovered that Rab11a protein was upregulated in 57/122 NSCLC tissues. Rab11a overexpression associated with advanced TNM stage, positive nodal status and poor patient prognosis. Rab11a overexpression promoted proliferation, colony formation, invasion and migration with upregulation of cyclin D1, cyclin E, and downregulation of p27 in NSCLC cell lines. Nude mice xenograft demonstrated that Rab11a promoted *in vivo* cancer growth. Importantly, we found that Rab11a induced YAP protein and inhibited Hippo signaling. Depletion of YAP abolished the effects of Rab11a on cell cycle proteins and cell proliferation. Furthermore, immunoprecipitation showed that Rab11a interacted with YAP in lung cancer cells. In conclusion, the present study suggestes that Rab11a serves as an important oncoprotein and a regulator of YAP in NSCLC.

## INTRODUCTION

Lung cancer is the leading cause of cancer-related death worldwide [1–3]. Non-small cell lung cancer (NSCLC) accounts for 85% of lung cancer cases. Despite recent advances in diagnosis and treatment including surgery, chemotherapy, radiotherapy and targeted therapy, the prognosis of NSCLC patient remains poor [4–10]. It is necessary to identify new molecular markers involved in the regulation of lung cancer cell aggressiveness.

Rab11a was originally identified as a vesicle trafficking protein [11], which could control the sensing of the relative levels of Rac activity [12]. Rab11a contributes to mitotic spindle organization/orientation and endosome recycling to the membrane [13, 14]. Active Rab11a is required for E-cadherin trafficking and lumen formation during epithelial morphogenesis [15]. Active Rab11a can carry E-cadherin to the cell-cell contacts while inactive Rab11a fails to regulate E-cadherin membrane recycling process [16]. Most studies concerning Rab11a focused on its role on vesicle transportation. The expression and function of Rab11a in human cancers have not been well investigated. There is one study showing that active Rab11a could induce the colorectal cell transformation and migration [17]. Rab11a was also reported to activate Wnt signaling in human pancreatic cancers [18]. To data, there was no report concerning protein expression pattern and clinical significance of Rab11a in human non-small cell lung cancer. In addition, the mechanism of Rab11a in cancer proliferation and invasion remains unclear.

The Hippo pathway controls tissue growth and tumorigenesis by inhibiting cell proliferation and promoting apoptosis [19, 20]. In mammals, activated Lats1/2 phosphorylates and inactivates the downstream effector YAP by sequestering them in the cytoplasm and degrading them. Without inhibition through Hippo signaling, YAP translocates into the nucleus, binding to transcription factors TEAD and induces transcription activation of target genes such as CTGF and enhances cell proliferation [21]. YAP nuclear localization and YAP/TEAD luciferase reporter activity are used to reflect Hippo signaling activity.

In this study, we examined Rab11a protein in 122 NSCLC tissues and analyzed its clinical significance. We also investigated its biological function and association with Hippo signaling pathway in NSCLC cell lines.

## RESULTS

### High expression of Rab11a associates with advanced stage of NSCLC

**Figure 1:**
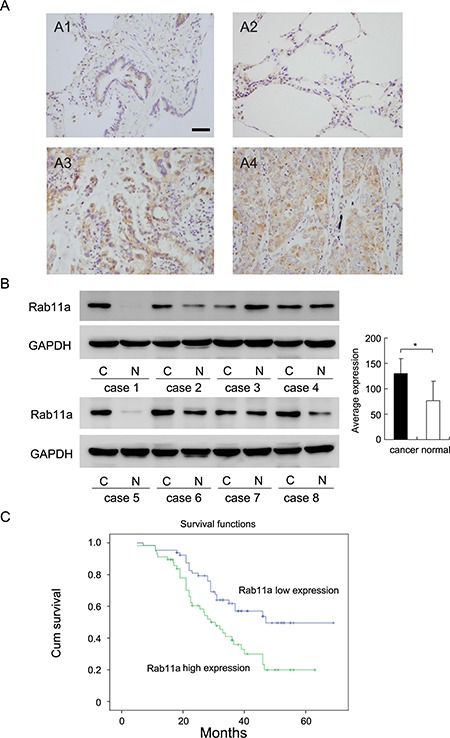
Expression of Rab11a in non-small cell lung cancers (**A**) Negative staining of Rab11a in normal bronchial epithelial cells and alveolar cells (A1&A2). Strong staining of Rab11a in lung squamous cell carcinoma(A3) and adenocarcinoma(A4). (Magnification 200×) (Bar indicates 50 μm) (**B**) Rab11a protein expression in 8 cases of lung cancer tissues and corresponding normal tissues. (**C**) Survival analysis of patients with Rab11a expression and those without. The overall survival was significantly lower in patients with Rab11a- high expression NSCLCs than in patients with Rab11a-low expression NSCLCs.

We investigated Rab11a expression in 122 NSCLC tissue specimens and 26 normal lung tissue specimens by immunohistochemistry. Rab11a protein was mainly located in the cytoplasmic compartment of tumor cells. In 26 normal lung tissues, weak/negative cytoplasmic staining (final score < 4) was observed in normal bronchial epithelial cells and alveolar cells (Figure 1A1). As for lung cancer tissues, 46.72% (57/122) cases showed high cytoplasmic Rab11a staining(final score ≥ 4) (Figure 1A2 and 1A3) and the rest showed low Rab11a staining. We examined the correlation between Rab11a status and clinical factors. We found that Rab11a overexpression significantly associated with advanced TNM stage (Table 1, *p* = 0.0015) and positive nodal status (*p* = 0.0027). No difference was observed in the Rab11a status according to the age, gender, histology, differentiation and tumor size (Table 1).

**Table 1:** Distribution of Rab11a status in NSCLC according to clinicopathological characteristics

Characteristics	Number of patients	Rab11a low expression	Rab11a high expression	*P*
Age				
< 60	61	32	29	0.8560
≥ 60	61	33	28	
Gender				
Male	73	38	35	0.7409
Female	49	27	22	
Differentiation				
Well	40	22	18	0.7901
Moderate-Poor	82	43	39	
Histology				
Adenocarcinoma	69	35	34	0.5188
Squamous cell carcinoma	53	30	23	
TNM stage				
I	55	38	17	0.0015
II+III	67	27	40	
Tumor status				
T1	47	28	19	0.2699
T2–T4	75	37	38	
Nodal metastasis				
Negative	73	47	26	0.0027
Positive	49	18	31	

We also examined Rab11a protein in 8 cases of fresh lung cancer tissues with their corresponding normal tissue. As shown in Figure 1B, Rab11a was overexpressed in 5 of 8 tissues examined. We analyzed the grey value of western blot bands and found that average expression of Rab11a in cancer tissues was significantly higher than that in normal tissues (Student's *t* test, *p <* 0.05).

Kaplan-Meier survival analysis showed significantly decreased overall survival in patients with high Rab11a compared with those with positive expression (*p* = 0.004, Log-Rank test; Figure 1C). In addition, univariate analysis showed that TNM stage and Rab11a status were both significant prognostic factors (TNM stage: hazard ratio, 2.370, *p <* 0.001; Rab11a status: hazard ratio, 1.458, *p* = 0.003). Multivariate analysis using a Cox regression model indicated that TNM stage was an independent, unfavorable prognostic factor (hazard ratio, 2.205, *p <* 0.001, Table 2).

**Table 2:** Univariate and multivariate analysis for predictive factors in patients with NSCLC

	Univariate		Multivariate	
Factors	Hazard ratio(95% CI)	*p* value	Hazard ratio(95% CI)	*p* value
Histology	1.168 (0.711–1.919)	0.5396	1.309 (0.776–2.208)	0.3131
Differentiation	1.683 (0.955–2.966)	0.0716	1.413 (0.936–2.133)	0.0998
Age	1.121 (0.686–1.832)	0.6479	0.930 (0.550–1.572)	0.7861
Gender	0.885 (0.534–1.469)	0.6372	0.938 (0.553–1.590)	0.8118
TNM Stage	2.370 (1.697–3.310)	< 0.001	2.232 (1.541–3.233)	< 0.001
Rab11a	1.458 (1.137–1.870)	0.003	1.235 (0.932–1.637)	0.1412

### Rab11a overexpression promotes proliferation, migration and invasion of lung cancer cells

**Figure 2:**
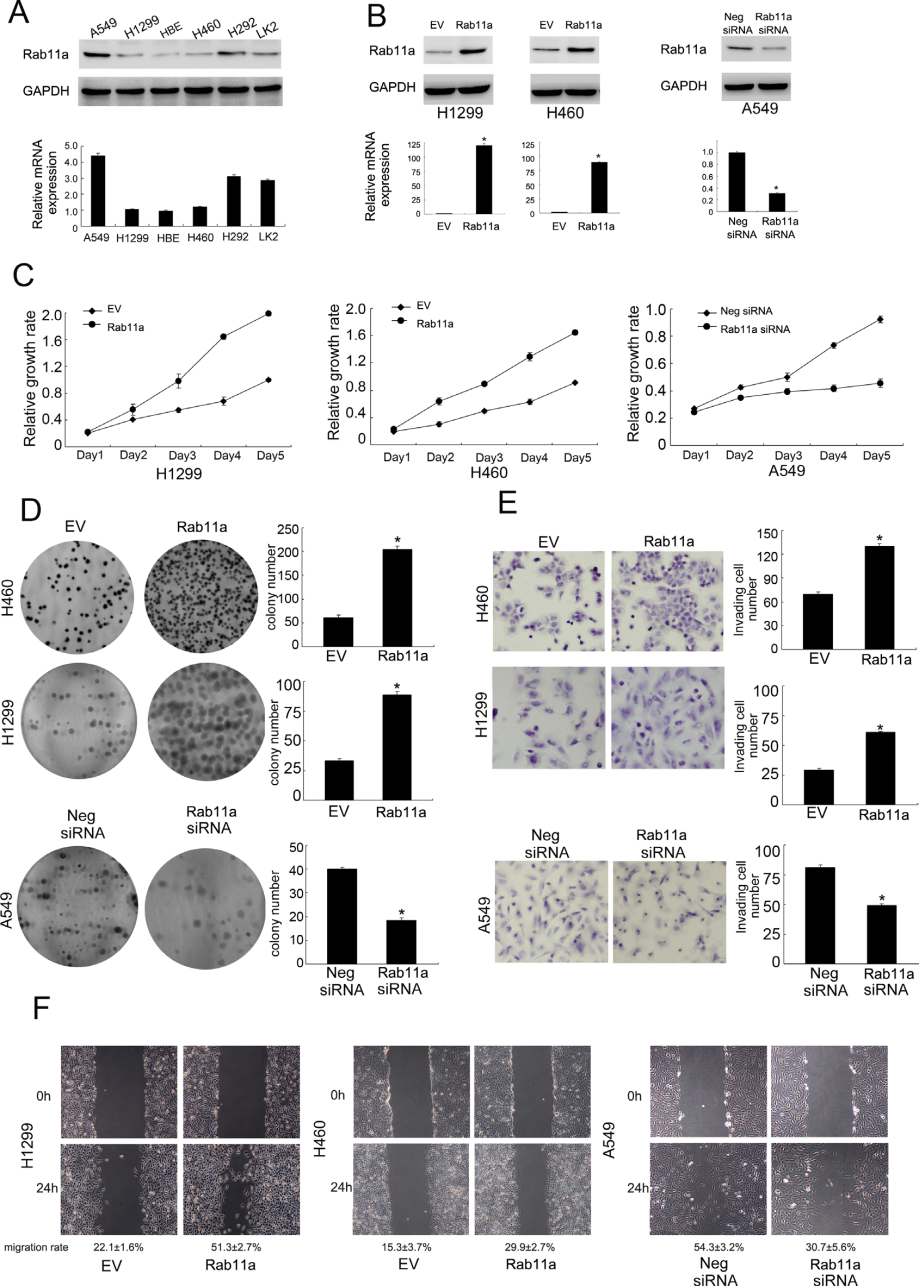
Rab11a expression in lung cancer cell lines and its role on proliferation, invasion and migration (**A**) Endogenous expression of Rab11a was examined in HBE and lung cancer cell lines by western blot and RT-qPCR. Lung cancer cell lines showed significant upregulated Rab11a expression. (**B**) Western blot and RT-qPCR analysis showed that pCMV6-Rab11a plasmid markedly increases its levels in H460 and H1299 cells compared with control. Rab11a plasmid downregulated its expression in A549 cells. (**C**) MTT showed that Rab11a overexpression in H1299 and H460 cells greatly promoted the proliferation rate while Rab11a depletion inhibited proliferation rate. (**D**) Rab11a overexpression in H1299 and H460 cells promoted the colony formation ability while Rab11a depletion inhibited colony formation ability. (**E**) Rab11a overexpression in H1299 and H460 cells greatly promoted cell invasion while Rab11a depletion inhibited invading ability of A549 cells. (**F**) Wound healing assay demonstrated that Rab11a overexpression increased cell migration in H1299 and H460 cell lines while its depletion downregulated A549 cell migration.

Relative expression level of Rab11a was analyzed by western blot in a panel of lung cancer cell lines. In accordance with immunohistochemical results, Rab11a protein expression was remarkably increased in 3/5 NSCLC cell lines (A549, H292 and LK2) compared with normal HBE cell line (Figure 2A). H1299 and H460 cell lines were selected for Rab11a plasmid transfection. siRNA was used in A549 cell line. Overexpression and knocking down efficiency was confirmed by western blot and RT-qPCR analysis (Figure 2B). MTT assay and colony formation assay were carried out to examine its role on cancer cell growth. As shown in Figure 2C and 2D, Rab11a overexpression greatly promoted the proliferation rate (Day 5, H1299 EV vs. Rab11a: 0.99 ± 0.02 vs. 1.98 ± 0.03, *p <* 0.05; H460 EV vs. Rab11a: 0.91 ± 0.01 vs. 1.64 ± 0.04, *p <* 0.05, Figure 2C) and the potential of colony formation (H1299 EV vs. Rab11a: 33.3 ± 1.8 vs. 88.6 ± 3.1, *p <* 0.05; H460 EV vs. Rab11a: 61.3 ± 5.1 vs. 204.3 ± 7.2, *p <* 0.05, Figure 2D), while Rab11a depletion in A549 cells inhibited proliferation (Day 5, A549 Neg siRNA vs. Rab11a siRNA: 0.92 ± 0.02 vs. 0.45 ± 0.03, *p <* 0.05) and colony formation ability (A549 Neg siRNA vs. Rab11a siRNA: 40.2 ± 0.5 vs. 18.3 ± 1.2, *p <* 0.05). To characterize the effect of Rab11a on cell invasion and migration, matrigel invasion assay and wound healing assay were performed. As shown in Figure 2E, significant increased invading ability was observed in cells with Rab11a transfection compared with empty controls (H1299 EV vs. Rab11a: 29.2 ± 1.4 vs. 61.3 ± 0.6, *p <* 0.05; H460 EV vs. Rab11a: 70.1 ± 2.3 vs. 130.5 ± 3.3, *p <* 0.05, Figure 2E). Rab11a depletion in A549 cells reduced invading ability (A549 Neg siRNA vs. Rab11a siRNA: 81.2 ± 2.1 vs. 49.5 ± 1.3, *p <* 0.05). Wound healing assay demonstrated that Rab11a overexpression increased cell migration in H1299 and H460 cell lines while its depletion downregulated A549 cell migration (*p <* 0.05). The rate of migration distance was calculated and presented in Figure 2F.

### Rab11a facilitates cell cycle and regulates cell cycle related proteins

**Figure 3:**
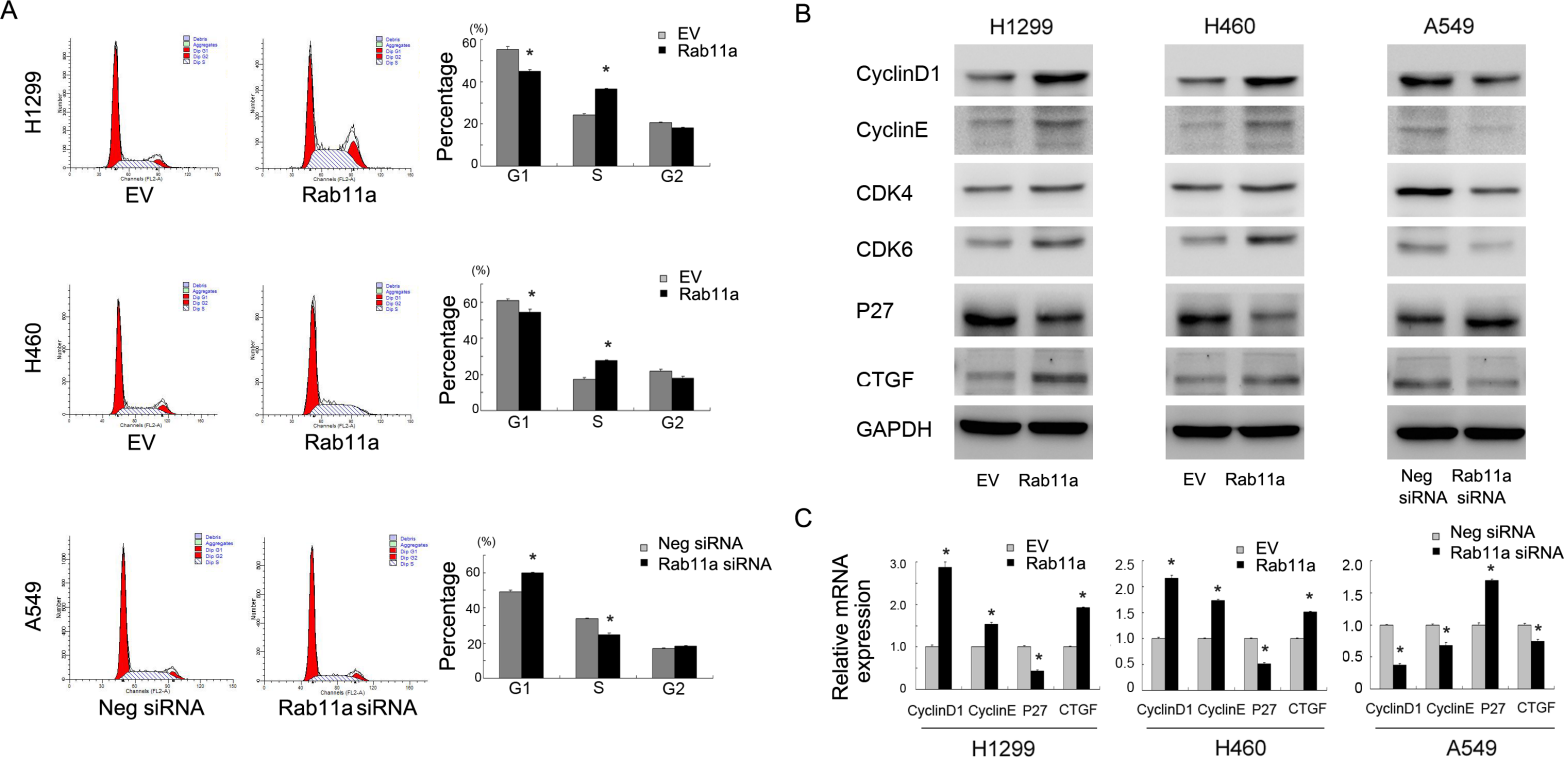
Rab11a promotes cell cycle progression and related proteins (**A**) Cell cycle analysis showed that Rab11a transfection decreased cell percentage in G1phase and increased the cell percentage in S phase. Rab11a depletion exhibited the opposite effects. (**B**) Western blot analysis showed that Rab11a overexpression increased the protein expression of cyclin D1, cyclin E, CDK4/6, CTGF and decreased p27 expression. Rab11a depletion showed the opposite effects in A549 cells. (**C**) Realtime PCR analysis showed that Rab11a overexpression increased the mRNA expression of cyclin D1, cyclin E, CTGF and decreased p27. Rab11a depletion showed the opposite effects. *p < 0.05.

The aforementioned results indicate that Rab11a leads to increased cellular proliferation and invasion. We checked the effect of Rab11a on cell cycle progression. As shown in Figure 3A, Rab11a overexpression induced G1-S transition in H460 and H1299 cell lines. Rab11a-siRNA inhibited cell cycle progression in A549 cell line. To underline the possible mechanisms, we examined a panel of growth and invasion related proteins. As shown in Figure 3B, Rab11a transfection significantly increased cell cycle protein cyclin D1, cyclin E, CDK4, CDK6 and reduced expression of cell cycle inhibitor p27. Rab11a depletion in A549 cells showed the opposite effects. We also found that Rab11a could induce CTGF while Rab11a depletion reduced its expression. Using Realtime RT-PCR, we found that Rab11a positively regulated mRNA expression of cyclin D1, cyclin E, CTGF and negatively regulated p27 in both H1299 and H460 cell lines. Rab11a depletion downregulated cyclin D1, cyclin El CTGF mRNA while upregulated p27 mRNA expression Figure 3C.

### Rab11a induces YAP nuclear localization and inhibits Hippo signaling

**Figure 4:**
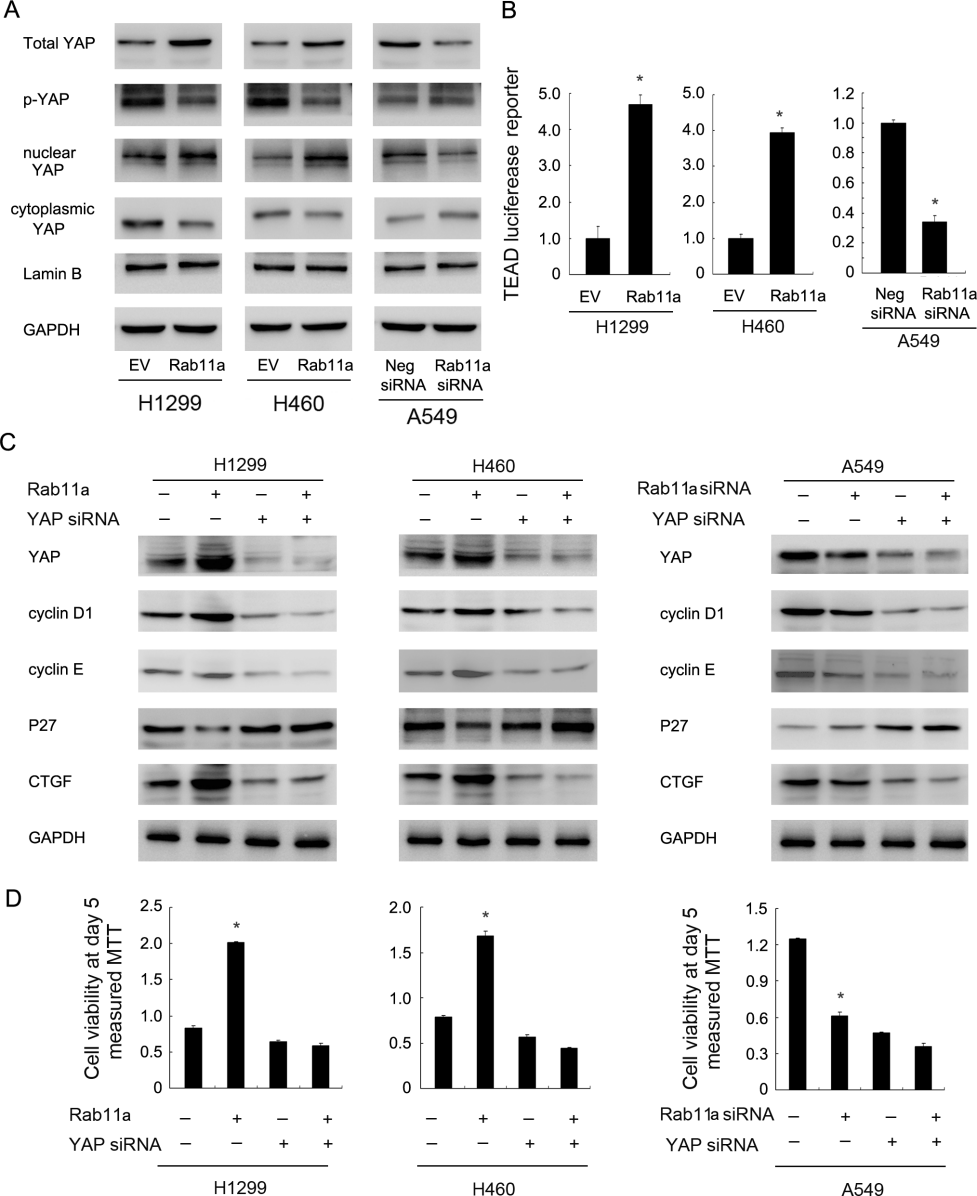
Rab11a regulates NSCLC proliferation and cell cycle proteins through upregulation of YAP (**A**) Rab11a overexpression in H1299 and H460 cell lines resulted in upregulation of total YAP and nuclear YAP protein. Rab11a depletion decreased YAP protein. (**B**) Rab11a overexpression could activate the luciferase activity of TEAD reporter plasmid in H1299 and H460 cell lines. YAP depletion in A549 cell line showed the opposite effects. (**C**) YAP siRNA treatment abolished the effect of Rab11a overexpression or depletion on CTGF and cell cycle proteins cyclin D1, cyclin E and p27 in A549, H1299, H460 cells. (**D**) MTT showed that YAP siRNA abolished the growth promoting effect of Rab11a in lung cancer cells. *p < 0.05.

CTGF was reported as a downstream target of Hippo pathway. To determine whether Rab11a is involved in the regulation of Hippo signaling in lung cancer cells, we examined the level of YAP and its nuclear localization in Rab11a transfected and depleted cells. The data showed that Rab11a overexpression in H1299 and H460 cell lines resulted in upregulation of total YAP protein. Rab11a depletion decreased YAP protein (Figure 4A). Consistently, western blot using nuclear proteins showed the level of nuclear YAP was downregulated after siRNA treatment and Rab11a transfection upregulated YAP nuclear localization (Figure 4A). The level of p-YAP was slightly decreased by Rab11a transfection.

Nuclear/cytoplasmic distribution and phosphorylation of YAP significantly influence its stabilization and downstream signaling. To test whether Rab11a could regulate Hippo signaling, we transfection lung cancer cells with Rab11a plasmid and siRNA, together the with TEAD reporter plasmid which reflect activation of YAP target genes transcription. We observed that the Rab11a overexpression could activate the luciferase activity of TEAD reporter plasmid (Figure 4B). Therefore, Rab11a might stabilize and upregulate YAP protein and thus increase the transcription of target genes, leading to inhibition of Hippo signaling (Figure 4B).

Next, we asked whether YAP mediates Rab11a induction of CTGF and cell cycle proteins. As shown in Figure 4C, the YAP siRNA was employed to downregulate endogenous YAP expression. cyclin proteins and CTGF were inhibited and p27 was upregulated by YAP-siRNA. In YAP-siRNA treated lung cancer cells, the Rab11a siRNA induced change of CTGF and cell cycle proteins was not significant. YAP siRNA treatment also abolished the effect of Rab11a overexpression on CTGF and cell cycle proteins in H1299 and H460 cells (Figure 4C). In addition, YAP siRNA significantly reduced the effect of Rab11a on cell proliferation (Figure 4D). Importantly, the effect of Rab11a siRNA or plasmid was not obvious in cells with YAP siRNA treatment compared with control cells. Together, these results suggested that Rab11a regulates lung cancer progression through, at least partly, regulation of YAP and inhibition of Hippo signaling pathway.

### Rab11a interacts with YAP and promotes lung cancer cell growth *in vivo*

**Figure 5:**
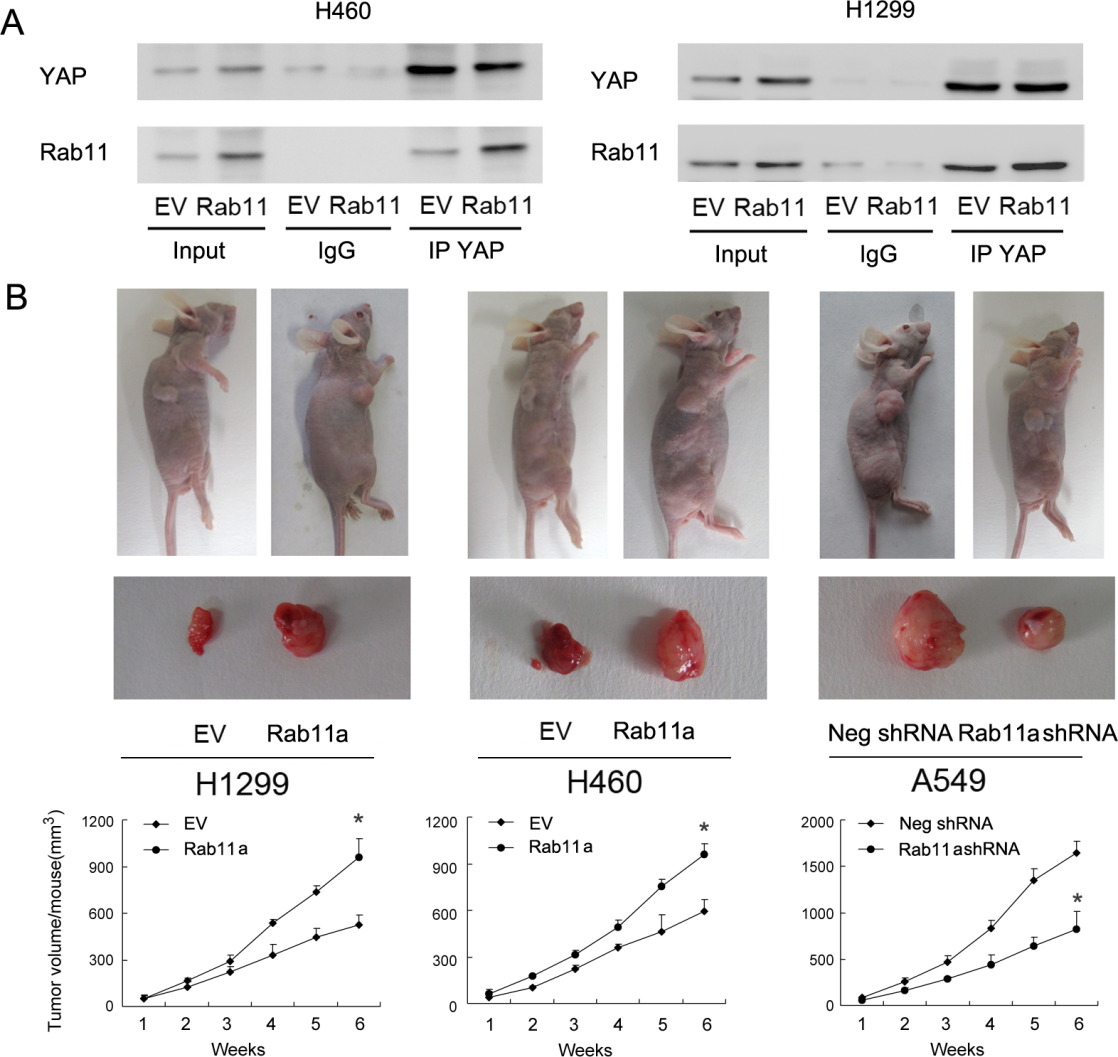
Rab11a interacts with YAP and promotes lung cancer cell growth *in vivo* (**A**) Rab11a and YAP co-immunoprecipitated in H1299 and H460 cells, with or without Rab11a overexpression. We did not detect their association when extracts were immunoprecipitated with normal rabbit IgG. (**B**) The rate of tumor growth of Rab11a overexpressed stable H1299 and H460 cell lines is higher compared with that of the control plasmid-transfected cells Rab11a-depleted stable A549 cell line showed slower growth speed compared with control. *p < 0.05.

To explore the possible mechanism of Rab11a induced YAP upregulation, co-immunoprecipitation was performed to examine if there is an association between Rab11a and YAP protein. We immunoprecipitated YAP from cell lysates and analyzed them by western blotting for Rab11a conjugation. As shown in Figure 5A, Rab11a and YAP co-immunoprecipitated in both H1299 and H460 cells with or without Rab11a overexpression. To examine its effect on *in vivo* tumor growth in nude mice, Rab11a-shRNA stable trasnfected A549 cells and Rab11a overexpressed stable H1299 and H460 cell lines were established, which were established by G418 selection. The volume of xenograft tumor of Rab11a overexpressed stable cells increased compared with that of the control plasmid-transfected cells (Figure 5B). On the other hand, Rab11a-depleted A549 cells showed decreased growth speed compared with negative control.

## DISCUSSION

During the last decade, advances have been made in the understanding of the Hippo signaling pathway. However, less is known about the function of the Hippo signaling pathway in mammals, particularly in the development of human cancers. In this study, we were able to show that Rab11a overexpression facilitated lung cancer cell growth and invasion. In addition, Rab11a upregulated lung cancer growth through YAP protein. To date, this is the first report concerning the relationship between Rab family protein and Hippo signaling in human cancers.

We first showed that Rab11a protein was upregulated in NSCLC specimen, which correlated with nodal metastasis and TNM stage. Importantly, high expression of Rab11a correlated with poor prognosis. Accordingly, Rab11a levels in lung cancer cell lines were higher than that in normal bronchial cell line HBE. Previous reports indicate that Rab11a serves as a cancer biomarker in pancreatic and breast cancers [18, 22]. Our clinical data is in accordance with these studies, demonstrating that Rab11a contributes to the malignant progression of NSCLC.

Next, we showed that Rab11a overexpression significantly promoted *in vitro* and *in vivo* cell growth, which was dependent on its role of cell cycle induction. Rab11a overexpression upregulated cyclin D1, cyclin E, CTGF and downregulated p27. CTGF (Connective tissue growth factor) ranks among top upregulated genes by Hippo effector YAP. It is reported that CTGF could lead to the upregulation of cyclin proteins and downregulation of p27 expression [23, 24]. These data suggest that Rab11a regulate aggressive behavior of NSCLC possibly through regulation of CTGF.

YAP is the main transducer of Hippo signaling which induces target genes including CTGF, resulting in cell proliferation and apoptosis evasion [25, 26]. Our previous studies demonstrated that YAP overexpression promoted lung cancer cell growth, invasion and correlated with poor patient survival [27]. In this study, we showed that Rab11a overexpression increased YAP expression and its nuclear localization. Conversely, Rab11a siRNA downregulated YAP and decreased its nuclear localization. In addition, YAP phosphorylation was also inhibited by Rab11a. Using luciferase reporter, we found that Rab11a overexpression upregulated transcription activity of YAP/TEAD. Dephosphorylated YAP translocates into the nucleus and binds to TEAD proteins, which activates downstream target such as CTGF and promotes proliferation [28, 29]. Furthermore, using siRNA, we were able to demonstrated that the biological effects of Rab11a was dependent on YAP. Taken together, our observations demonstrate that Rab11a regulate lung cancer aggressiveness through regulation YAP and Hippo signaling pathway.

Using immunoprecipitation, we found that Rab11a could interact with endogenous YAP. This interaction was stronger in cells transfected with Rab11a plasmid, which further validates the link between Rab11a and Hippo signaling in NSCLC. Since Rab11a is a vesicle trafficking protein which regulates turnover of many intracellular proteins including vascular endothelial-cadherin, protease-activated receptor-1 and PAR3 [30–32], we believe that Rab11a could also interacts and regulates the turnover of YAP, inhibiting its degradation, which induce its nuclear accumulation and inhibition of Hippo signaling.

In conclusion, this study delineates the functional role of Rab11a in lung cancer progression. Rab11a expression in lung cancer specimens may be a valuable biomarker for aggressive behavior and poor prognosis. We also revealed that Rab11a inhibits Hippo signaling and promotes non-small cell lung cancer cell proliferation and invasion through regulation of YAP. Rab11a might be a potential target for the therapeutic strategy in NSCLC.

## MATERIALS AND METHODS

### Tissue samples

The study was approved by the ethical committee of China Medical University. 122 cases of NSCLC samples were obtained from the the first affiliated hospital of china medical university since 2007 to 2010. All procedures performed in studies involving human participants were in accordance with the ethical standards of the institutional and/or national research committee and with the Helsinki declaration and its later amendments or comparable ethical standards. Informed consent was obtained from all individual participants included in the study. All patients underwent curative surgical resection without prior chemotherapy or radiation therapy. 26 normal lung tissue specimens were obtained from non-cancerous lung diseases. In addition, 8 paired fresh specimens including both tumor tissue and corresponding normal tissue were stored at −70°C immediately after resection for extraction of protein.

### Immunohistochemistry

The tissue sections were treated with xylene, graded alcohol. Antigen retrieval was performed in 0.01 M citrate buffer. H2O2 was used for blockage. Sections were treated with goat serum for 20 minutes. Then the slides was incubated Rab11a antibody (1:300 dilution, Proteintech, USA) overnight at 4°C. EliVision Super Kit (Maixin, Fuzhou, China) was then used for immunostaining. All tumor slides were examined randomly by two independent pathologists. Five views were selected in the center of tumor slides for evaluation. Rab11a staining was located in the cytoplasmic compartment of tumor cells. Immunostaining of Rab11a was scored following a semi-quantitative scale by evaluating the intensity and percentage of cells. The intensity of staining score was indicated as 0 (no staining), 1 (weak staining), 2 (strong staining). Staining percentage was scored as 0 (0%), 1 (1–25%), 2 (26–50%), 3 (51–75%) and 4 (76–100%). Each score was multiplied to a final score of 0 to 8. Rab11a status was regarded as low Rab11a expression (score < 4) or high expression/overexpression (score ≥ 4).

### Cell culture and transfection

Human NSCLC cell lines H292, LK2, H460, A549, H1299 and normal bronchial epithelial cell line HBE were purchased from ATCC. RPMI-1640 (Gibco, USA) with 10% FBS was used for cell culture. pCMV6-Rab11a plasmid and the control empty vector pCMV6 were obtained from Origene (Origene, USA). Transfection of plasmid (0.12 μg per well for 6-well plate) was performed using Attractene reagent (Qiagen, Germany). Rab11a siRNA and control siRNA were obtained from Dharmacon (GE healthcare, USA). Transfection of siRNA (0.2 nmol per well for 6-well plate) was performed using Dharmafect reagent (GE healthcare, USA). Rab11a (sh)RNA was designed using the rnaidesigner (Invitrogen, USA): shRab11a, 5′-GAATGTCAGACAGACGCGAAAATT-3′. Vectors encoding shRNA was generated using pENTR/U6. shRNA plasmids using Attractene following the manufacturer's instructions. The empty plasmid was used as a negative control. Selection was accomplished with G418 (Sigma) at a concentration of 0.5 mg/mL.

### Western blotting and immunoprecipitation

Total proteins from cell lines or fresh lung cancer/normal tissues were extracted in lysis buffer and quantified using the Bradford method. 30 mg protein was separated by SDS-PAGE. Nuclear/ cytoplasmic protein were separated using NE-PER Nuclear and Cytoplasmic Extraction Reagents (Thermo scientific, USA) according to the manufacturer's protocol. Samples were transferred to PVDF membranes (Millipore, Billerica, MA, USA) and incubated overnight at 4°C with primary antibodies. Antibodies against cyclin D1, cyclin E, p27, CDK4, CDK6, CTGF, p-YAP, YAP (1:1000 dilution) and GAPDH (1:2000 dilution) were obtained from Cell Signaling Technology (Beverly, MA, USA). Rab11a antibody was from Proteintech (Proteintech, USA). After incubation with HRP-coupled anti-mouse or rabbit IgG antibody at 37°C for 2 hours. Target proteins on PVDF membrane were visualized using Pierce ECL kit and captured using a DNR BioImaging System (DNR, Jerusalem, Israel).

For immunoprecipitation, Magnetic Beads (Bio-Rad SureBeads) were incubated with antibodies and unbound antibodies were washed away. Then beads-antibody complex was incubated with target protein. The beads were magnetized using SureBeads magnetic rack and supernatant was discarded. Then elution buffer was used to collect purified target protein for western blot analysis.

### RNA extraction and real-time RT-PCR

Total RNA was isolated from cultured cells using the RNAiso Plus reagent (TaKaRa, Dalian, China). Reverse transcription of RNA was performed using the PrimeScript RT Mastermix (TaKaRa, Dalian, China). Quantitative real-time PCR was performed using SYBR mastermix (TaKaRa, Dalian, China) on the 7900HT fast Real-time PCR system (Applied Biosystems). The fold change of gene expression was calculated by the 2^–ΔΔCt^ Method. Experiments were repeated in triplicate. The primer sequences are listed as follows: Rab11a forward, 5′-AAA GCAAGAGCACCATTGGAG-3′, Rab11a reverse, 5′-TG CCCTGCTGTGTCCCAT-3′; cyclin D1 forward, 5′-TGGA GGTCTGCGAGGAACA-3′, cyclin D1 reverse, 5′-TTCAT CTTAGAGGCCACGAACAT-3′; cyclin E forward, 5′-AG CCAGCCTTGGGACAATAAT-3′, cyclin E reverse, 5′-GA GCCTCTGGATGGTGCAAT-3′; p27 forward, 5′-CTGC AACCGACGATTCTTCTACT-3′, p27 reverse, 5′-CTTCT GAGGCCAGGCTTCTT-3′; CTGF forward, 5′-GTTAC CAATGACAACGCCTCCT-3′, CTGF reverse, 5′-TGCA CTTTTTGCCCTTCTTAATGT-3′; β-actin forward, 5′-CCT GAACCCCAAGGCCAAC-3′, β-actin reverse, 5′-GAT AGCACAGCCTGGATAGCAAC-3′.

### Colony formation and MTT assays

For colony formation assay, cells were plated into three 6-cm cell culture dishes (1000 cells), cultured for 2 weeks in medium. Then plates were washed with PBS and stained with Giemsa. Colony number was manually counted.

For MTT assay, cells were plated in 96-well plates about 2000 cells per well and cultured for 5 days. 20 μl of MTT (thiazolyl blue) solution was added per well and plates were incubated for 4 hours in a incubator. The medium was removed and 150 μl of DMSO was added to each well. The plate was measured at a wavelength of 490 nm.

### Matrigel invasion assay

Matrigel invasion assay was carried out using a 24-well Transwell chamber from Costar (Corning, USA) coated with 20 μl Matrigel with a dilution rate of 1:6 (BD Bioscience, USA). 48 hours after the transfection, cells were trypsinized and transferred to the upper chamber with our serum and incubated for 18 hours. Lower chamber was added with medium supplemented with 10% serum. Non-invaded cells were wiped out and cells invaded through the filter were fixed with 4% paraformaldehyde and stained with hematoxylin.

### Wound healing assay

After 24 h of growth, cells were seeded into 6-well tissue culture plates at a density of about 80–90% confluence as a monolayer. The monolayer was gently and slowly scratched using a 1 ml pipette tip under aseptically conditions. The detached cells were removed and washed with PBS, then incubated in serum for the indicated times. Photos of the stained monolayer were taken under a microscope. The gap distance was quantitatively evaluated using Image J software.

### Flow cytometry for cell cycle analysis

Cells were seeded into 6 cm tissue culture dishes. Forty eight hours after transfection, cells were harvested, fixed in 1% paraformaldehyde, washed with PBS and stained with 5 mg/ml propidium iodide in PBS supplemented with RNase A (Roche, Indianapolis, IN) for 30 minutes at room temperature. Cells in each individual phase of the cell cycle were determined based on their DNA ploidy profile.

### *In vivo* xenograft tumor models

BALB/c athymic nude mice (4–5 weeks old) were purchased from Shanghai Slac Laboratory Animals Ltd. (Shanghai, China) and housed in the Laboratory Animal Center of China Medical University (Shenyang, China). All animal experiments and procedures conformed to the institutional animal care guidelines. A xenograft model of human lung cancer was established by subcutaneous right armpit injections of stable cell line (1–2 * 10^6^). Tumor size was measured every 4–7 d. Following 2 months growth, animals were sacrificed, and tumors removed. Experiment was performed in triplicate.

### Luciferase reporter assay

To assess change of Hippo signaling and YAP transcription activity, we performed luciferase assay according to the manufacturer's protocol (Promega, USA). Cells were transfected with luciferase reporter plasmid 8xGTIIC-luciferase with Rab11a plasmid/siRNA. Cells were transfected with 8xGTIIC-luciferase reporter along with the Renilla luciferase reporter, which was used for normalization. The luciferase activity was measured in cellular extracts using a dual luciferase reporter gene assay kit (Promega, San Luis Obispo, CA, USA).

### Statistical analysis

SPSS version 16 for Windows was used for all statistical analyses. A chi-square test was used to examine possible correlations between Rab11a expression and clinicopathologic factors. The Kaplan-Meier method was used to estimate the probability of patient survival. Differences in the survival of subgroups of patients were compared by using Mantel's log-rank test. The Cox regression model was used for multivariate analysis. Student's *t-test* was used to compare densitometry data on focus numbers between control and transfected cells. All *p* values are based on a two-sided statistical analysis, and *p <* 0.05 was considered to indicate statistical significance.
